# Impact of Health Behavior Practices on Emergency Department Utilization Among Older Adults With Diabetes

**DOI:** 10.1093/geroni/igaf122.2390

**Published:** 2025-12-31

**Authors:** Suhyun Kim, Ye-Ji Lim

**Affiliations:** Kyungpook National University, Daegu, Republic of Korea; Kyungpook National University Hospital, Daegu, Republic of Korea

## Abstract

**Background:**

Older adults with diabetes are at heightened risk for emergency department (ED) visits due to disease-related complications. Identifying modifiable factors, such as health behaviors, that contribute to ED utilization is crucial for developing targeted interventions to enhance self-management and reduce healthcare burdens.

**Objective:**

This study examines the association between demographic and disease characteristics, health behavior practices, and ED visits among older adults aged 65 years and older diagnosed with diabetes.

**Methods:**

A descriptive correlational study was conducted using data from 956 older adults diagnosed with diabetes, drawn from the Korean Healthcare Panel Annual Data (2014–2017). Data analysis was performed using descriptive statistics, Mann-Whitney U tests, chi-square analysis, and univariate regression analysis in SPSS 26.0.

**Results:**

The findings revealed that older age, activity limitations due to disability, depression, and a greater number of chronic comorbidities significantly increased the likelihood of ED visits among older adults with diabetes. Additionally, fewer days of moderate physical activity and walking were associated with a higher risk of ED utilization.

**Conclusion:**

Promoting sustained physical activity is essential in mitigating ED visits among older adults with diabetes. Healthcare providers should implement tailored interventions that enhance mobility and address psychological and physical health barriers to improve self-care management and reduce acute healthcare utilization.

**Implications for Clinical Practice:**

Integrating physical activity counseling into routine care, alongside comprehensive chronic disease management strategies, may help prevent ED visits and improve overall well-being in this population.

